# Well-being in amyotrophic lateral sclerosis: a pilot experience sampling study

**DOI:** 10.3389/fpsyg.2014.00704

**Published:** 2014-07-08

**Authors:** Ruben G. L. Real, Thorsten Dickhaus, Albert Ludolph, Martin Hautzinger, Andrea Kübler

**Affiliations:** ^1^Department of Psychology I, Institute of Psychology, University of WürzburgWürzburg, Germany; ^2^Weierstrass Institute for Applied Analysis and Stochastics, Research Group “Stochastic Algorithms and Nonparametric Statistics”Berlin, Germany; ^3^Department of Neurology, University of UlmUlm, Germany; ^4^Department of Clinical Psychology and Psychotherapy, University of TübingenTübingen, Germany; ^5^Institute for Medical Psychology and Behavioural Neurobiology, University of TübingenTübingen, Germany

**Keywords:** amyotrophic lateral sclerosis, ALS, coping, well-being, experience sampling, ESM, reminiscence, rumination

## Abstract

**Objective:** The aim of this longitudinal study was to identify predictors of instantaneous well-being in patients with amyotrophic lateral sclerosis (ALS). Based on flow theory well-being was expected to be highest when perceived demands and perceived control were in balance, and that thinking about the past would be a risk factor for rumination which would in turn reduce well-being.

**Methods:** Using the experience sampling method, data on current activities, associated aspects of perceived demands, control, and well-being were collected from 10 patients with ALS three times a day for two weeks.

**Results:** Results show that perceived control was uniformly and positively associated with well-being, but that demands were only positively associated with well-being when they were perceived as controllable. Mediation analysis confirmed thinking about the past, but not thinking about the future, to be a risk factor for rumination and reduced well-being.

**Discussion:** Findings extend our knowledge of factors contributing to well-being in ALS as not only perceived control but also perceived demands can contribute to well-being. They further show that a focus on present experiences might contribute to increased well-being.

## 1. Introduction

Amyotrophic Lateral Sclerosis (ALS) is a rare neurodegenerative disease characterized by progressive paralysis (Logroscino et al., 2008). In the absence of a cure (Logroscino et al., 2008, 2010) the treatment of ALS focuses on the alleviation of symptoms and the maintenance of the best possible degree of functioning (Clarke et al., 2001). A rich literature exists on factors influencing quality of life and coping with the disease (c.f. Pagnini, 2013). For example, different styles of coping (Matuz et al., 2010; Montel et al., 2012a), spirituality (McLeod and Clarke, 2007), or care-giver relations (Chiò et al., 2004) have all been related to psychosocial well-being in patients with ALS. In line with psychological theories on subjective quality of life (e.g., Rapkin and Schwartz, 2004) the great physical burden of ALS shows no simple relationship to well-being, with many studies showing small and insignificant correlations (Robbins et al., 2001; Pagnini, 2013) or even positive associations (Lulé et al., 2009). However, many of the questionnaires used in research on well-being and quality of life in patients with ALS require participants to generate aggregate statements about their experiences, e.g., by asking them how they felt (on average) during the past days (Cohen et al., 1995; Robbins et al., 2001) or even weeks (Hammer et al., 2008). Thus, little is known about the factors which are associated with instantaneous well-being, i.e., well-being as it fluctuates from moment to moment throughout the day.

In this study, therefore, the experience sampling method (ESM) was used (Larson and Csikszentmihalyi, 1983; Stone et al., 2003) to analyze possible predictors of well-being. Experience sampling, originally developed because of concerns of how accurate people are in “reconstructing their experience[s] after the fact” (Larson and Csikszentmihalyi, 1983 p. 42), asks participants to record theirdemands and control experiences directly at the moment of sampling. By randomizing sampling times throughout the study period, it is possible to collect representative data on the participants' experiences, such as well-being, and possible influencing factors (Shiffman et al., 2008; Scollon et al., 2009).

At its most basic level, flow theory assumes that the physical and mental activities people engage in, influence their well-being. More specifically, it predicts that well-being is highest if perceived control and perceived demands match in a given situation (Csikszentmihalyi, 1990). Any imbalance between demands and abilities results in reduced well-being, e.g., feelings of frustration, when demands exceed abilities, or of boredom, when abilities exceed situational demands (Csikszentmihalyi, 1990). Given that the progressive loss of motor functions requires constant adaptation and change (King et al., 2009), we hypothesized that finding this balance would be of crucial importance in patients with ALS. A second prediction of flow theory is that well-being depends on the extent to which thoughts are focused on present experiences (Schmidt et al., 2007). However, the little data available from patients with ALS is heterogeneous. Support comes from a study by Plahuta et al. (2002), which found that hopelessness, a future oriented direction of thought, is related to increased suffering and higher intentions of suicide in patients with ALS. However, the use of the coping strategy “planning” (Carver, 1997), an arguably more direct and less negatively valenced indicator of “thoughts directed toward the future” does not seem to be strongly related to well-being (Montel et al., 2012b). Qualitative findings from interviews with patients are also mixed (Fanos et al., 2008). On the one hand, several subjects indicated the use of “living in the moment” as a successful coping strategy while others sought consolation in remembering positive past experiences. The constant adaptation and change required for coping with ALS (King et al., 2009), necessitates disengaging from goals no longer attainable, or anticipating disengagement from goals which may become unattainable in the future. Generally, confrontation with unattainable goals—even more so when the factors which make a goal unattainable are essentially uncontrollable as is the case in ALS (Nolen-Hoeksema et al., 2008)—has been shown to be a risk factor for rumination, and, thus, decreased well-being (Nolen-Hoeksema, 1991; Martin and Tesser, 1996). Therefore, we hypothesized that thinking about the past and future would be associated with reduced well-being, and that rumination would mediate this effect.

## 2. Methods

### 2.1. Patients

A convenience sample of ten patients (seven male; see Table 1) fulfilling the revised El Escorial criteria for clinically definite ALS (Brooks et al., 2000) was recruited via the Institute for Medical Psychology and Behavioral Neurobiology of the University of Tübingen and the Department of Neurology of the University of Ulm. All participants contacted for inclusion agreed to participate in this study. Mean time since diagnosis was 38.10 months (*SD* = 35.96, range = 12–129). One patient had a percutaneous endoscopic gastrostomy (PEG), and three were non-invasively ventilated. The study was approved by the Ethical Review Board of the Medical Faculty, University of Tübingen. Written informed consent was obtained from all participants.

**Table 1:** **Patients' Characteristics**.

	**ALSFRS-R**	**ADI-12**	**well-being**	***M***	***SD***	**Range**
Age (years)	0.52	0.32	−0.09	52.30	11.56	35–71
ALSFRS-R		0.59^#^	−0.75^*^	19.20	10.57	1–35
ADI-12			−0.63^#^	20.70	7.41	12–34
well-being				4.79	0.69	3.93–5.86

Intercorrelations, means, and standard correlations. Note: N = 10.

# p < 0.10,

* p < 0.05.

### 2.2. Procedure

We collected data on well-being, perceived demands, perceived control, temporal direction of thoughts and rumination over the course of two weeks at three randomly chosen times per day using the ESM. Patients were provided with a pager that beeped when they should record their experiences using a standardized questionnaire. To avoid interference with morning and evening caring routines, sampling was restricted to between 10 a.m. and 6 p.m. Patients were instructed to answer the ESM-questionnaires immediately after the beep and while answering the questions to refer only to the situation in which the beep occurred. At the beginning of the two weeks study period, patients were visited at their homes and the procedure was explained in full detail and questions concerning the study protocol were answered.

### 2.3. Measures

Functional status was assessed with the revised ALS Functional Rating Scale (ALSFRS-R) (Cedarbaum et al., 1999), and the extent of depressive symptoms with the ALS specific ALS Depression Inventory (ADI-12) (Hammer et al., 2008). These standardized measures were collected once at the beginning of the two week study period.

The ESM-questionnaire used in this study was previously used by Csikszentmihalyi and Larson (1987) and assessed the extent of perceived demands and control with two items, “How challenging was your activity?” and “Was the situation under your control?” To minimize participants' burden in terms of time and effort, rumination was measured with the single item “Did your thoughts turn round in circles?” (Jong-Meyer et al., 2009) and temporal direction of thoughts with two face-valid items (“Did you look ahead/to the past?”). Each item was scored on a 10-point Likert-type scale ranging from 0 (low) to 9 (high). Patients' well-being was measured with 13 well-being-related items developed by Larson and Csikszentmihalyi (1983). Each item was scored on a 7-point Likert-type scale, and a composite score reflecting well-being was derived by averaging ESM-items of the well-being category. To determine the reliability of this scale while taking the inter-correlation of repeated measurements into account Cronbach's α was calculated across subjects for every time point during the 2-week study period, yielding 42 coefficients. Reliability of the well-being scale was satisfactory (median α = 0.86).

Patients completed an average of 33.30 assessments (range 13–43). Fifty percent of all questionnaires were answered within 10 min and 75% within 45 min after the beep. Further analysis revealed that without the most disabled participant (one male who used a personal computer to answer the questionnaires) 75% of all questionnaires were answered within 30 min after the beep. One patient was excluded from the following analyses because of excessive numbers of missing items, leading to a final sample size of *N* = 9.

### 2.4. Statistical analyses

Random intercept multiple regression analysis was used to test the hypothesis that well-being depended on the balance of demands and control (Edwards and Cooper, 1990; Edwards, 1994 also see Supplementary Material). The analysis was also performed with functional status included as a covariate, which indicated that the results we report here were independent of functional status. Mediation analysis (MacKinnon et al., 2002; Kenny et al., 2003) was used to test the hypothesis that thinking about the past would have a negative effect on well-being and that this effect would be mediated by rumination. We report 95% confidence intervals for the mediated effect (Tofighi and MacKinnon, 2011). The same analysis was repeated for thinking about the future. Analyses were performed in R (R Development Core Team, 2011). Cohen's *f*^2^ was calculated as a measure of the effect size (Selya et al., 2012).

## 3. Results

Average well-being (see Table 1) was marginally negatively associated with symptoms of depression, but significantly positively associated with functional status. Functional status was marginally positively associated with symptoms of depression. Patient's age was not associated with functional status, symptoms of depression, or well-being.

Our first hypothesis suggested that well-being would depend on the balance of perceived control and demands. However, no support was found for this prediction (see Supplementary Material). Instead, results indicated that perceived control was associated with increased well-being (*b* = 0.18, *SE* = 0.03, *p* < 0.001, *f*^2^ = 0.22), regardless of the level of perceived demands. In contrast, the effect of perceived demands depended on whether these demands were perceived as controllable: Only, if demands were perceived as *controllable*, they were associated with increased well-being (*b* = 0.09, SE = 0.02, *p* < 0.001, *f*^2^ = 0.17). If demands were perceived as *uncontrollable*, the positive effect of demands was significantly reduced (*b* = −0.14, *SE* = 0.05, *p* < 0.01, *f*^2^ = 0.03), rendering the effect of demands on well-being non-significant (*b* = 0.02, *SE* = 0.05, *p* > 0.70, *f*^2^ = 0.01).

Our second hypothesis proposed that thinking about the past would lead to increased rumination which in turn would reduce well-being. Table 2 shows that thinking about the past (Row 1), thinking about the future (Row 2), and rumination (Row 3) were associated with lower well-being, and that thinking about the past (Row 4) was significantly and thinking about the future (Row 5) was marginally associated with higher rumination. Mediation analysis confirmed a negative effect of thinking about the past on well-being via rumination (*b* = −0.03, 95% CI [−0.05 −0.02]). No evidence was found that the negative effect of thinking about the future on well-being was mediated via increased rumination (*b* = −0.01, 95% CI [−0.02 0.001]).

**Table 2:** **Results of mediation analysis**.

	**Predicted**	**Predictor**	***b***	***SE***	***DF***	***t***	***p***	***f^2^***
**PREDICTORS OF WELL-BEING**								
1	Well-being	Thinking about the past	−0.03	0.01	250.83	−2.73	0.01	0.02
2	Well-being	Thinking about the future	−0.03	0.01	250.97	−2.08	0.04	0.01
3	Well-being	Rumination	−0.08	0.01	255.84	−5.60	<0.001	0.10
**PREDICTORS OF RUMINATION**								
4	Rumination	Thinking about the past	0.41	0.04	252.89	10.34	<0.001	0.40
5	Rumination	Thinking about the future	0.09	0.05	252.50	1.70	0.09	0.01
**MEDIATION MODELS**								
6	Well-being	Thinking about the past	0.00	0.01	247.77	0.17	0.87	
7		Rumination	−0.08	0.02	254.12	−4.79	<0.001	
8	Well-being	Thinking about the future	−0.02	0.01	250.30	−1.66	0.10	
9		Rumination	−0.08	0.01	254.83	−5.43	<0.001	

## 4. Discussion

Little is known about how patients with ALS cope with their disease from moment to moment in daily life. Thus, in this study the ESM was used to record patients' experiences throughout the day. Despite their severe physical disability, patients were able to complete the ESM-questionnaires within a short time after the scheduled signal. These results provide evidence that even a potentially demanding procedure such as the ESM may be a valuable tool in assessing patients' thoughts, well-being and cognitive state. The relationships between functional status, presence of depressive symptoms and well-being indicated that depressive symptoms were more present and well-being was lower in early stages of the disease. This result mirrors findings of better quality of life in patients who are longer affected by the disease (Lulé et al., 2008, 2009).

Flow theory predicts that well-being depends on finding the optimal balance between perceived demands and control (Csikszentmihalyi, 1990), and we hypothesized that finding this balance would be of crucial importance in patients with ALS. However, our data did not support this hypothesis. Instead, but in line with previous findings (Plahuta et al., 2002), perceived control was associated with increased well-being, regardless of the level of perceived demands. However, our results extend this idea, by showing that increased demands can also contribute to increased well-being, as long as they are perceived as controllable. While flow theory is primarily concerned with experiences related to activities in the present, experiences may also differ in their temporal orientation. We hypothesized that if attention is not focused on the here and now, but directed toward the past or the future, this would increase the risk for rumination, which in turn would reduce well-being. Our results indicate that thinking about the past was indeed associated with decreased well-being and that this effect was mediated by rumination. While some patients may find solace in remembering the good times they have had (Fanos et al., 2008), reminiscing may also introduce the risk of being reminded of unattainable goals, thus, in turn, increasing the risk of reduced well-being. Likewise, thinking about the future was negatively associated with well-being; however, it was only loosely related to rumination, and no support was found for rumination mediating a negative effect of thinking about the future on well-being. We may thus speculate that thoughts about the future are not necessarily ruminative, possibly only in patients experiencing hopelessness (Plahuta et al., 2002).

### 4.1. Limitations and considerations for future studies

#### 4.1.1. Sampling

Several limiting factors of the current study deserve attention. To minimize interruption of daily routine, time slots in which patients had pre-set appointments (e.g., occupational therapy) were excluded from the experience sampling and sampling was restricted to the time between 10 a.m. and 6 p.m. This restriction may have prevented us from sampling several activities of daily living, e.g., personal hygiene, which may be very demanding and frustrating for severely ill patients (Foley et al., 2014).

#### 4.1.2. Economic considerations

From a research perspective it is often desirable to use longer rather than shorter instruments, if only to increase reliability (Spearman, 1910). However, in a repeated measures context, this desire needs to be balanced with economic considerations, especially in a sample in which motor difficulties, e.g., when holding a pen, are common. Thus, to avoid spurious results, e.g., low well-being due to the frustrating experience of having to repeatedly answer a long questionnaire, and to reduce the risk of high levels of non-reponse (Iglesias and Torgerson, 2000) the questionnaires we used were kept as short as possible. Yet it is clear, that assessing rumination with only one item is a simplification of a complex construct. However, both empirical (Jong-Meyer et al., 2009) as well as theoretical accounts (Carver, 1996) suggest, that the item we used possesses high face validity for assessing rumination.

#### 4.1.3. Sample size and generalizability

Finally, although not uncommon in ESM studies (e.g., Teuchmann et al., 1999), the small sample size limits generalization of results, until future studies can replicate our findings. Further, our analyses focused on within-subject variables, but between-subject factors and their interactions could also play an important role. For example, care-givers tend to underestimate the patient's quality of life (Trail et al., 2003), and it might be interesting to know, whether this is accompanied by a tendency to shield the patient from demanding activities, which according to our findings, might actually improve the patient's well-being. To the best of our knowledge, our study is the first to employ the ESM in patients with ALS. Thus, little information on appropriate sample sizes was available beforehand, demonstrating the pilot character of this study. Judging from the obtained effect sizes, mainly small to medium according to Cohen (1992), we suggest increasing sample sizes in future studies. Results from simulation studies (Snijders and Bosker, 1993) suggest, that an increase in the number of participants might be of greater interest than increasing the number of observations per participant. Further, if a research question focused primarily on between-subject factors (see above) a larger sample size would also be recommended. Thus, it should be kept in mind, that the optimal balance between number of participants and the number of observations per participant is dependent on the particular research question.

The ESM methodology has been applied in a variety of chronic diseases, e.g., in patients with cancer, chronic pain, cardiovascular diseases, or patients requiring hemodialysis (c.f. Smyth and Stone, 2003), testifying to the wide applicability of the method (c.f. Christensen et al., 2003). However, depending on the research questions, sophisticated data analysis methods, and technical equipment, e.g., electronic paging and recording devices with a user-friendly and unobstrusive interface, may be needed. Given the large challenge of living with ever progressing motor impairments, we suggest that future studies may take full advantage of the widespread availability of smartphones which lend themselves to ESM-studies (e.g., Runyan et al., 2013). Using such a system it might even become possible to densely track the evolution of coping with ALS over prolonged periods of time. However, apart from generating valuable data for research purposes, such a procedure might also affect patients' well-being, e.g., by making relations between certain situations and well-being transparent to the patient.

### 4.2. Summary

Our results suggest that the ESM may be a valuable tool to elucidate components of coping and well-being in daily life in patients with ALS. Perceived control over challenging situations seems to be of major importance for maintaining high well-being. We further show that well-being may not only depend on perceived control, but that it also depends on the level of perceived demands. Encouraging patients to pursue, within limits, potentially demanding activities might help to improve their well-being.

Both thoughts directed toward the past and the future may negatively affect well-being, whereas rumination mediates this effect when thoughts are directed toward the past. We speculate that patients who experience poor well-being and may even be depressed might benefit from interventions which not only strengthen an internal locus of control (Nonnenmacher et al., 2013; Foley et al., 2014) but also encourage living in the present.

On a more global perspective the flow-theoretical approach and our results correspond well with recent work aiming at evaluating mindfulness-based (Kocovski et al., 2009) interventions in patients with ALS (Pagnini et al., 2014a,b). These interventions aim to help focusing on the experiences in the here and now, and thus to reduce the risk of reactive, e.g., ruminative, thoughts, which may otherwise reduce well-being or even lead to depression (Bishop et al., 2004). Our results provide further evidence for this promising approach, by highlighting the importance of the temporal direction of thoughts and the importance of perceived demands and control for the experience of well-being in patients with ALS.

## Funding

Funding for this study was provided by a grant to Ruben G. L. Real of the research training group 1253/1 which is supported by the German Research Foundation (Deutsche Forschungsgemeinschaft, DFG) by Federal and Länder funds. The study sponsor had no role in the study design, collection, analysis or interpretation of the data, writing the manuscript, or the decision to submit the paper for publication.

### Conflict of interest statement

The authors declare that the research was conducted in the absence of any commercial or financial relationships that could be construed as a potential conflict of interest.

## References

[B1] Bishop S. R. Lau M. Shapiro S. Carlson L. Anderson N. D. Carmody J. (2004). Mindfulness: a proposed operational definition. Clin. Psychol. Sci. Pract. 11, 230–241 10.1093/clipsy.bph077

[B2] Brooks B. R. Miller R. G. Swash M. Munsat T. L. for the World Federation of Neurology Research Group on Motor Neuron Diseases. (2000). El escorial revisited: revised criteria for the diagnosis of amyotrophic lateral sclerosis. Amyotroph. Lateral Scler. Other Motor Neuron Disord. 1, 293–299 10.1080/146608200300079536 11464847

[B3] Carver C. S. (1996). Goal engagement and the human experience, in Ruminative Thoughts, Volume IX of *Advances in Social Cognition*, ed Wyer R. S. (Mahwah, NJ: Lawrence Erlbaum), 49–59

[B4] Carver C. S. (1997). You want to measure coping but your protocol's too long: consider the brief cope. Int. J. Behav. Med. 4, 92–100 10.1207/s15327558ijbm0401_6 16250744

[B5] Cedarbaum J. M. Stambler N. Malta E. Fuller C. Hilt D. Thurmond B. (1999). The alsfrs-r: a revised als functional rating scale that incorporates assessments of respiratory function. J. Neurol. Sci. 169, 13–21 10.1016/S0022-510X(99)00210-5 10540002

[B6] Chiò A. Gauthier A. Montuschi A. Calvo A. Di Vito N. Mutani R. (2004). A cross sectional study on determinants of quality of life in als. J. Neurol. Neurosurg. Psychiatry 75, 1597–1601 10.1136/jnnp.2003.033100 15489393 PMC1738825

[B7] Christensen T. Barrett L. Bliss-Moreau E. Lebo K. Kaschub C. (2003). A practical guide to experience-sampling procedures. J. Happiness Stud. 4, 53–78 10.1023/A:1023609306024

[B8] Clarke S. Hickey A. O'Boyle C. Hardiman O. (2001). Assessing individual quality of life in amyotrophic lateral sclerosis. Qual. Life Res. 10, 149–158 10.1023/A:1016704906100 11642685

[B9] Cohen J. (1992). A power primer. Psychol. Bull. 112, 155–159 10.1037/0033-2909.112.1.155 19565683

[B10] Cohen S. R. Mount B. M. Strobel M. G. Bul F. (1995). The mcgill quality of life questionnaire: a measure of quality of life appropriate for people with advanced disease. a preliminary study of validity and acceptability. Palliative Med. 9, 207–219 10.1177/026921639500900306 7582177

[B11] Csikszentmihalyi M. (1990). Flow: The Psychology of Optimal Experience. New York, NY: Harper and Row

[B12] Csikszentmihalyi M. Larson R. (1987). Validity and reliability of the experience-sampling method. J. Nerv. Ment. Dis. 175, 526–536 10.1097/00005053-198709000-00004 3655778

[B13] Edwards J. R. (1994). The study of congruence in organizational behavior research: critique and a proposed alternative. Organ. Behav. Hum. Decis. Process. 58, 51–100 10.1006/obhd.1994.1029

[B14] Edwards J. R. Cooper C. L. (1990). The person-environment fit approach to stress: recurring problems and some suggested solutions. J. Organ. Behav. 11, 293–307 10.1002/job.4030110405

[B15] Fanos J. H. Gelinas D. F. Foster R. S. Postone N. Miller R. G. (2008). Hope in palliative care: From narcissism to self-transcendence in amyotrophic lateral sclerosis. J. Palliative Care 11, 470–475 10.1089/jpm.2007.0098 18363490

[B16] Foley G. Timonen V. Hardiman O. (2014). Exerting control and adapting to loss in amyotrophic lateral sclerosis. Soc. Sci. Med. 101, 113–119 10.1016/j.socscimed.2013.11.003 24560231

[B17] Hammer E. V. Häcker S. Hautzinger M. Meyer T. D. Kübler A. (2008). Validity of the als-depression-inventory (adi-12): a new screening instrument for depressive disorders in patients with amyotrophic lateral sclerosis. J. Affect. Disord. 109, 213–219 10.1016/j.jad.2007.11.012 18262283

[B18] Iglesias C. Torgerson D. (2000). Does length of questionnaire matter? a randomised trial of response rates to a mailed questionnaire. J. Health Serv. Res. Policy 5, 219–221 10.1177/135581960000500406 11184958

[B19] Jong-Meyer R. D. Parthe T. Projektgruppe (2009). Einfluss von achtsamkeitsübung und dezentrierung auf rumination und spezifität autobiographischer erinnerungen. Z. Klin. Psychol. Psych. 38, 240–249 10.1026/1616-3443.38.4.240

[B20] Kenny D. A. Bolger N. Korchmaros J. D. (2003). Lower level mediation in multilevel models. Psychol. Methods 8, 115–128 10.1037/1082-989X.8.2.115 12924810

[B21] King S. J. Duke M. M. O'Connor B. A. (2009). Living with amyotrophic lateral sclerosis/motor neurone disease (als/mnd): decision-making about ‘ongoing change and adaptation’. J. Clin. Nurs. 18, 745–754 10.1111/j.1365-2702.2008.02671.x 19239541

[B22] Kocovski N. L. Segal Z. V. Battista S. R. (2009). Mindfulness and psychopathology: problem formulation, in Clinical Handbook of Mindfulness, ed Didonna F. (New York, NY: Springer New York), 85–98

[B23] Larson R. Csikszentmihalyi M. (1983). The experience sampling method, in Naturalistic Approaches to Studying Social Interaction, Vol. 15, ed Reis H. T. (San Francisco, CA: Jossey-Bass), 41–46

[B24] Logroscino G. Traynor B. J. Hardiman O. Chiò A. Couratier P. Mitchell J. D. (2008). Descriptive epidemiology of amyotrophic lateral sclerosis: new evidence and unsolved issues. J. Neurol. Neurosurg. Psychiatry 79, 6–11 10.1136/jnnp.2006.104828 18079297

[B25] Logroscino G. Traynor B. J. Hardiman O. Chio A. Mitchell D. Swingler R. J. (2010). Incidence of amyotrophic lateral sclerosis in europe. J. Neurol. Neurosurg. Psychiatry 81, 385–390 10.1136/jnnp.2009.183525 19710046 PMC2850819

[B26] Lulé D. Häcker S. Ludolph A. Birbaumer N. Kübler A. (2008). Depression and quality of life in patients with amyotrophic lateral sclerosis. Deutsch. Ärztebl. Int. 105, 397–403 10.3238/arztebl.2008.0397 19626161 PMC2696844

[B27] Lulé D. Zickler C. Häcker S. Bruno M. A. Demertzi A. Pellas F. (2009). Life can be worth living in locked-in syndrome. Prog. Brain Res. 177, 339–351 10.1016/S0079-6123(09)17723-3 19818912

[B28] MacKinnon D. P. Lockwood C. M. Hoffman J. M. West S. G. Sheets V. (2002). A comparison of methods to test mediation and other intervening variable effects. Psychol. Methods 7, 83–104 10.1037/1082-989X.7.1.83 11928892 PMC2819363

[B29] Martin L. L. Tesser A. (1996). Some ruminative thoughts, in Ruminative Thoughts, Vol. IX of *Advances in Social Cognition*, ed Wyer R. S. (Mahwah, NJ: Lawrence Erlbaum), 1–47

[B30] Matuz T. Birbaumer N. Hautzinger M. Kübler A. (2010). Coping with amyotrophic lateral sclerosis: an integrative view. J. Neurol. Neurosurg. Psychiatry 81, 893–898 10.1136/jnnp.2009.201285 20587497

[B31] McLeod J. E. Clarke D. M. (2007). A review of psychosocial aspects of motor neurone disease. J. Neurol. Sci. 258, 4–10 10.1016/j.jns.2007.03.001 17445834

[B32] Montel S. Albertini L. Spitz E. (2012a). Coping strategies as related to medical and demographic data in amyotrophic lateral sclerosis. Acta Neurol. Scand. 125, 136–141 10.1111/j.1600-0404.2011.01513.x 21470190

[B33] Montel S. Albertini L. Spitz E. (2012b). Coping strategies in relation to quality of life in amyotrophic lateral sclerosis. Muscle Nerve 45, 131–134 10.1002/mus.22270 22190320

[B34] Nolen-Hoeksema S. (1991). Responses to depression and their effects on the duration of depressive episodes. J. Abnorm. Psychol. 100, 569–582 10.1037/0021-843X.100.4.569 1757671

[B35] Nolen-Hoeksema S. Wisco B. E. Lyubomirsky S. (2008). Rethinking rumination. Perspect. Psychol. Sci. 3, 400–424 10.1111/j.1745-6924.2008.00088.x 26158958

[B36] Nonnenmacher S. Hammer E. M. Lulé D. Hautzinger M. Kübler A. (2013). Psychische störungen und individuelle lebensqualität bei der chronisch progredient-terminalen erkrankung amyotrophe lateralsklerose (als). Z. Klin. Psychol. Psych. 42, 55–63 10.1026/1616-3443/a000186

[B37] Pagnini F. (2013). Psychological wellbeing and quality of life in amyotrophic lateral sclerosis: a review. Int. J. Psychol. 48, 194–205 10.1080/00207594.2012.691977 22731673

[B37b] Pagnini F. Di Credico C. Gatto R. Fabiani V. Rossi G. Lunetta C. (2014a). Meditation training for people with amyotrophic lateral sclerosis and their caregivers. J. Altern. Complement. Med. 20, 272–275 10.1089/acm.2013.0268 24328393 PMC3994974

[B39] Pagnini F. Phillips D. Langer E. (2014b). A mindful approach with end-of-life thoughts. Front. Psychol. 5:138 10.3389/fpsyg.2014.00138 24600429 PMC3930860

[B40] Plahuta J. M. McCulloch B. J. Kasarskis E. J. Ross M. A. Walter R. A. McDonald E. R. (2002). Amyotrophic lateral sclerosis and hopelessness: psychosocial factors. Soc. Sci. Med. 55, 2131–2140 10.1016/S0277-9536(01)00356-2 12409126

[B41] Rapkin B. D. Schwartz C. E. (2004). Toward a theoretical model of quality-of-life appraisal: implications of findings from studies of response shift. Health Qual. Life Out. 2:14 10.1186/1477-7525-2-14 15023229 PMC408464

[B42] R Development Core Team (2011). R: A Language and Environment for Statistical Computing. Vienna: R Foundation for Statistical Computing

[B43] Robbins R. A. Simmons Z. Bremer B. A. Walsh S. M. Fisher S. (2001). Quality of life in als is maintained as physical function declines. Neurology 56, 442–444 10.1212/WNL.56.4.442 11222784

[B44] Runyan J. D. Steenbergh T. A. Bainbridge C. Daugherty D. A. Oke L. Fry B. N. (2013). A smartphone ecological momentary assessment - intervention app for collecting real-time data and promoting self-awareness. PLoS ONE 8:e71325 10.1371/journal.pone.0071325 23977016 PMC3743745

[B45] Schmidt J. A. Shernof D. J. Csikszentmihalyi M. (2007). Individual and situational factors related to the experience of flow in adolescence, in Oxford Handbook of Methods in Positive Psychology, Series in Positive Psychology, eds Ong A. D. Van Dulmen M. H. M. (Oxford; New York, NY: Oxford University Press), 542–558

[B46] Scollon C. N. Prieto C. K. Diener E. (2009). Experience sampling: promises and pitfalls, strength and weaknesses, in Assessing Well-Being, Volume 39 of Social Indicators Research Series, ed Diener E. (Dordrecht: Springer), 157–180

[B47] Selya A. S. Rose J. S. Dierker L. C. Hedeker D. Mermelstein R. J. (2012). A practical guide to calculating cohen's f2, a measure of local effect size, from proc mixed. Front. Psychol. 3:111 10.3389/fpsyg.2012.00111 22529829 PMC3328081

[B48] Shiffman S. Stone A. A. Hufford M. R. (2008). Ecological momentary assessment. Annu. Rev. Clin. Psychol. 4, 1–32 10.1146/annurev.clinpsy.3.022806.091415 18509902

[B49] Smyth J. M. Stone A. A. (2003). Ecological momentary assessment research in behavioral medicine. J. Happiness Stud. 4, 35–52 10.1023/A:1023657221954

[B50] Snijders T. A. B. Bosker R. J. (1993). Standard errors and sample sizes for two-level research. J. Educ. Behav. Stat. 18, 237–259 10.3102/10769986018003237

[B51] Spearman C. (1910). Correlation calculated from faulty data. Br. J. Psychol. 3, 271–295

[B52] Stone A. A. Shiffman S. S. DeVries M. W. (2003). Ecological momentary assessment, in Well-Being, eds Kahneman D. Diener E. Schwarz N. (New York, NY: Russell Sage Foundation), 26–39

[B53] Teuchmann K. Totterdell P. Parker S. K. (1999). Rushed, unhappy, and drained: an experience sampling study of relations between time pressure, perceived control, mood, and emotional exhaustion in a group of accountants. J. Occup. Health Psychol. 4, 37–54 10.1037/1076-8998.4.1.37 10100112

[B54] Tofighi D. MacKinnon D. P. (2011). Rmediation: an r package for mediation analysis confidence intervals. Behav. Res. Methods 43, 692–700 10.3758/s13428-011-0076-x 21487904 PMC3233842

[B55] Trail M. Nelson N. D. Van J. N. Appel S. H. Lai E. C. (2003). A study comparing patients with amyotrophic lateral sclerosis and their caregivers on measures of quality of life, depression, and their attitudes toward treatment options. J. Neurol. Sci. 209, 79–85 10.1016/S0022-510X(03)00003-0 12686407

