# Supplementary material for: Well-being in amyotrophic lateral sclerosis: a pilot experience sampling study
Source: Front Psychol. 2014 Jul 8;5:704. doi: 10.3389/fpsyg.2014.00704 (PMC4085718; doi:10.3389/fpsyg.2014.00704)
Supplement: Supplementary file 1 [file Presentation1.PDF]

Frontiers in Psychology for Clinical Settings

Research Article
1 July 2014

Supplementary Material: Well-being in Amyotrophic Lateral Sclerosis: a pilot Experience Sampling Study

Ruben GL Real[1,*], Thorsten Dickhaus[2], Albert Ludolph[3], Martin Hautzinger[4] and Andrea Kübler[1,5]

[1] *Institute of Psychology, Department of Psychology I, University of Würzburg, Würzburg, Germany*

[2] *Weierstrass Institute for Applied Analysis and Stochastics, Research Group "Stochastic Algorithms and Nonparametric Statistics", Berlin, Germany*

[3] *Department of Neurology, University of Ulm, Ulm, Germany*

[4] *Department of Clinical Psychology and Psychotherapy, University of Tübingen, Tübingen, Germany*

[5] *Institute for Medical Psychology and Behavioural Neurobiology, University of Tübingen, Tübingen, Germany*

Correspondence*:
Ruben GL Real
Insitute of Psychology, University of Würzburg, Marcusstr. 9-11, 97070 Würzburg, Germany, ruben.real@uni-wuerzburg.de

Psychological issues in Amyotrophic lateral sclerosis

Flow theory predicts that well-being depends on finding the optimal balance between perceived demands and control. In the following we describe in some detail the methods we used to the test this hypothesis.

1 TESTING AN ABSOLUTE DIFFERENCE MODEL

The hypothesis that well-being would be highest when the demands associated with an activity were matched by one's abilities implies an absolute difference model ($|X - Y|$) such that deviations in either direction reduce well-being (see Figure 1).

Let the variables Z, X, Y, and ϵ represent well-being, perceived control, perceived demands, and a random error term. The absolute difference model may then be expressed using the following regression equation (**Edwards**, 1994, 2001):

$$Z = \beta_0 + \beta_1(1 - 2W)(X - Y) + \epsilon \tag{1}$$

where $W = 0$ if $X \geq Y$, else $W = 1$. The notion of absolute differences is captured by the interplay of the definition of W and the difference term (X-Y). If X is greater than or equal to Y, the difference (X-Y) is positive and 1-2W reduces to 1, leaving the difference term unchanged. However, if X is smaller than Y the difference is negative but 1-2W reduces to -1, reversing the sign of the difference term. Equation 1 may be expanded to

$$Z = \beta_0 + \beta_1 X - \beta_1 Y - 2\beta_1 WX + 2\beta_1 WY + \epsilon \tag{2}$$

Equation 2 may then be compared to a standard multiple regression equation with terms X, Y and the product terms WX and WY. Conceptually, the product terms serve to capture the moderating effect of the difference between control (X) and demands (Y) on the effect of control and demands. However, it has been shown that product terms may only be interpreted as interactions when the constituent terms of the interaction are included in the regression (**Cohen**, 1978; **Brambor et al.**, 2006). This means, to be able to interpret WX or WY as the moderating effects of W on X and Y, W would have to be included in the equation. Equation 2 does not include a term W, as it assumes W's regression weight to be zero thereby dropping the term from the equation. Further, the multiple occurrences of β_1 in Equation 2 reveal that all these regression weights are assumed to be the same. To be able to test this assumption, the regression weights for X, Y, WX, WY, and W in Equation 2 are set free,

$$Z = \beta_0 + \beta_1 X + \beta_2 Y + \beta_3 W + \beta_4 WX + \beta_5 WY + \epsilon \tag{3}$$

and Equation 3 may then be used to estimate the empirical relation between the predictors and well-being.

EXAMPLE

After fitting the model specified by Equation 3, the obtained coefficients are compared to the constraints as defined in Equation 2. The logic behind this step is best illustrated by an example: Imagine applying Equation 3 to some data yielded the following parameter estimates. $\beta_0 = 0$, $\beta_1 = 1$, $\beta_2 = -1$, $\beta_3 = 0$, $\beta_4 = -2$, $\beta_5 = 2$. Note that the following conditions hold for these fictitious estimates: All coefficients are different from zero but not β_3, b) coefficients β_1 and β_2 are of equal absolute magnitude but opposite sign, c) coefficients β_4 and β_5 are of equal absolute magnitude but opposite sign, and d) β_4 is twice the negative of β_1. Inserting these estimates into Equation 3 yields,

$$Z = 0 + 1X - 1Y + 0W - 2WX + 2WY + \epsilon$$

After rearranging and conversion:

$$Z = 0 + 0W + 1(X - Y) - 2W(X - Y) + \epsilon$$

Since the parameter for W is zero, we can eliminate it from the equation and rearrange the equation:

$$Z = 0 + 1(1 - 2W)(X - Y) + \epsilon$$

Comparing this equation with Equation 1 shows they are the same. Thus, the parameters in this example would support an absolute difference model.

Any set of empirical coefficients β_1, β_2, β_3, β_4, and β_5 supports an absolute difference model if the following conditions hold: a) all coefficients to be significantly different from zero but not β_3, b) coefficients β_1 and β_2 to be of equal absolute magnitude but opposite sign, c) coefficients β_4 and β_5 to be of equal absolute magnitude but opposite sign, and d) β_4 to be twice the negative of β_1.

Finally, Equation 3 can be slightly adapted by introducing a random intercept ξ_j per subject to account for multiple measurements per subject:

$$Z_{i,j} = \beta_0 + \xi_j + \beta_1 X_{i,j} + \beta_2 Y_{i,j} + \beta_3 W_{i,j} + \beta_4 W_{i,j} X_{i,j} + \beta_5 W_{i,j} Y_{i,j} + \epsilon_{i,j} \quad (4)$$

with measurements i nested in subjects j, X=control, Y=demands, Z=well-being, and W=0 if control exceeded demands, and W=1 if demands exceeded control. In thirty-nine measurements patients reported equal values of perceived demands and control. These ties were corrected by pseudo-randomly setting W_ij to 0 or 1, and all predictors were centered on their shared mean (**Edwards**, 1994). Linear contrasts were used to test our hypothesis.

Figure 1: Illustration of the hypothesized (Panel a) and empirical (Panel b) relationship between well-being, perceived control and perceived demands.

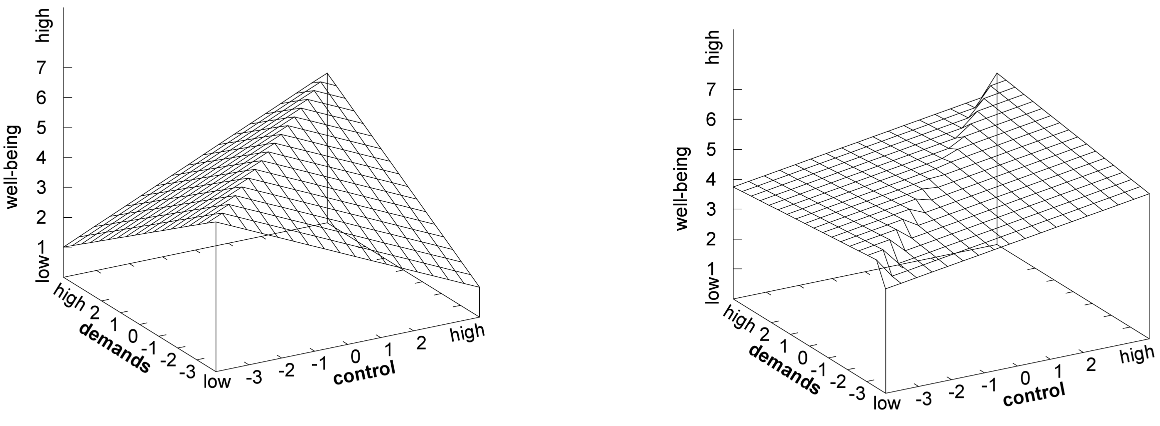

(a) hypothesized (b) empirical

Note. Panel a depicts the hypothesized relation between perceived control, perceived demands and well-being. Well-being is hypothesized to be highest, if perceived control and perceived demands are in balance. Panel b depicts the empirical relationship between these variables. If perceived control exceeds perceived demands, both, control and demands are associated with increased well-being . However, if perceived demands exceed perceived control increasing demands are associated with reduced well-being, while the positive association of perceived control with well-being remains unchanged.

2 RESULTS

Applying the model described in Equation 4 to our data indicated a significant fit for the whole model (AIC 288.35, BIC 315.53, $\chi^2 = 91.26, DF = 5, p < .001$). Provided that perceived control exceeded perceived demands, both, control and demand were associated with increased well-being ($b_1 = 0.18, SE = 0.03, p < .001$, $b_2 = 0.09, SE = 0.02, p < .001$). However, if demands exceeded perceived control increasing demands were associated with reduced well-being ($b_5 = -0.14, SE = 0.05, p < .01$), while the positive association of perceived control with well-being remained unchanged ($b_4 = -0.004, SE =$

$0.06, p > .93$). The effect of W, i.e. the point when demands begin to exceed control (see Equation 1) was not significantly different from zero ($b_3 = 0.6, SE = 0.10, p > .55$).

The first constraint required all coefficients but b_3 to be significant. Instead, only coefficients b_1, b_2 and b_5 were significant, while coefficient b_4 was not significant.

Formal testing of the model's other constraints showed that b_1 was not of equal magnitude but opposite sign as b_2, ($t(5) = 4.30, p < .01$), and b_4 was not twice the negative of b_1, ($t(5) = 3.02, p < .05$). However, coefficient b_4 was of equal magnitude but opposite sign as $b_5, t(5) = 1.26, p > .13$). Since all constraints have to be fulfilled simultaneously the pattern of coefficients did not support the idea of a necessary balance between situational demands and perceived control for optimal well-being.

REFERENCES

Brambor, T., Clark, W. R., and Golder, M. (2006), Understanding interaction models: Improving empirical analyses, *Political Analysis*, 14, 63–82

Cohen, J. (1978), Partialed products are interactions: Partialed powers are curve components, *Psychological Bulletin*, 85, 4, 858–866

Edwards, J. R. (1994), The study of congruence in organizational behavior research: Critique and a proposed alternative, *Organizational Behavior and Human Decision Processes*, 58, 51–100

Edwards, J. R. (2001), Ten difference score myths, *Organizational Research Methods*, 4, 265–287
